# ADAMTS-13 regulates neutrophil recruitment in a mouse model of invasive pulmonary aspergillosis

**DOI:** 10.1038/s41598-017-07340-3

**Published:** 2017-08-03

**Authors:** Astrid Alflen, Steve Prüfer, Katharina Ebner, Sebastian Reuter, Pamela Aranda Lopez, Inge Scharrer, Fumiaki Banno, Michael Stassen, Hansjörg Schild, Kerstin Jurk, Markus Bosmann, Hendrik Beckert, Markus P. Radsak

**Affiliations:** 1 IIIrd Dept. of Medicine, Johannes Gutenberg-University Medical Center, Johannes Gutenberg-University, Mainz, Germany; 2 Institute for Immunology, Johannes Gutenberg-University Medical Center, Johannes Gutenberg-University, Mainz, Germany; 3 Center for Thrombosis and Hemostasis, Johannes Gutenberg-University Medical Center, Johannes Gutenberg-University, Mainz, Germany; 4 Department of Molecular Pathogenesis, National Cerebral and Cardiovascular Center, Suita, Japan

## Abstract

Von Willebrand factor (VWF) is secreted as an acute phase protein during inflammation. ADAMTS-13 regulates the size and prothrombotic activity of VWF by it’s specific proteolytic activity. To determine the relevance of this regulatory pathway for the innate inflammatory response by polymorphonuclear neutrophils (PMN), we employed a mouse model of invasive pulmonary aspergillosis (IPA) where PMN functionality is crucial for fungal clearance and survival. IPA was induced by intratracheal application of *Aspergillus fumigatus* (*A*. *fumigatus*) conidia in wildtype (129/Sv/Pas) or ADAMTS-13 deficient (*Adamts13* ^−/−^) mice. While neutropenic mice developed lethal IPA, all wildtype mice survived the infection. In contrast to wildtype or VWF deficient mice, *Adamts13* ^−/−^ mice displayed more severe signs of disease with a lethal course in 24% with an increased fungal burden and signs of acute lung injury. Histology sections demonstrated a more pronounced perivascular leukocyte infiltration in support of a dysregulated inflammatory response in *Adamts13* ^−/−^ mice. Importantly, we observed no general defect in the activation of neutrophil functions in response to conidia or hyphae *in vitro*. Therefore, we conclude that the proteolytic regulation of VWF by ADAMTS-13 or ADAMTS-13 by itself is an important mechanism to control PMN recruitment in acute inflammatory processes, such as fungal pneumonias.

## Introduction

Invasive pulmonary aspergillosis (IPA) is a major threat to immunocompromised patients and caused by infection with the saprophytic fungus *Aspergillus fumigatus* (*A*. *fumigatus*). In particular, patients requiring immunosuppressive drugs for organ or allogeneic hematopoietic stem cell transplantation (HSCT) are at risk of developing IPA^1^. Despite the availability of potent antifungal drugs in clinical use, IPA remains a significant problem in the daily clinical patient care^2^. Neutropenia is one major risk factor for the development of IPA^3, 4^, emphasizing the critical role of polymorphonuclear neutrophils (PMN) in the innate immune response against fungal pathogens, while also monocytes are relevant in the regulation of antifungal immune responses^1^. PMN kill germinating *Aspergillus* conidia as well as hyphae^2^. While the size of hyphae may prevent phagocytosis by PMN, they are still in direct contact and cause hyphal damage both by oxidative and non-oxidative mechanisms. In this setting, the oxidative PMN effector functions are essential for survival of IPA^5^.

Von Willebrand factor (VWF) is produced as multimers of various sizes by endothelial cells and megakaryocytes and continuously released into the blood^6^. Ultra-large VWF multimers (UL-VWF) are stored in endothelial Weibel-Palade bodies or in alpha granules of platelets^7^ and can only be detected in the plasma after endothelial cell activation, i.e. in endotoxemia, thrombosis or thrombotic thrombocytopenic purpura (TTP)^8^. ADAMTS-13 (a disintegrin and metalloprotease with ThromboSpondin type 1 repeats-13) is produced by hepatic stellate cells^9^ as well as by endothelial cells^10^ and by megakaryocytes^11^ and responsible for cleaving UL-VWF preventing its pro-thrombotic activity^12^. In the pathogenesis of TTP, an inherited or acquired deficiency of ADAMTS-13 (mediated by inhibitory autoantibodies)^13, 14^ leads to thrombotic microangiopathy by the accumulation of UL-VWF triggering platelet and VWF rich thrombus formation, characteristic of this disease. Interestingly, extracellular DNA and myeloperoxidase as markers for PMN activation are detected in patients with active TTP^15^. In addition, increased levels of UL-VWF and decreased activity of ADAMTS-13 are also found in inflammatory conditions, such as endotoxemia and sepsis implicating a general role in inflammatory responses^16, 17^. VWF and platelets control PMN extravasation and recruitment to inflamed tissues by modulating vascular permeability, but not leukocyte rolling and adhesions, as shown in a mouse model of thioglycollate induced sterile peritonitis^18^.

The immune paralysis as in sepsis increases the susceptibility for IPA^19^, but the underlying mechanisms are currently not clear. Hence, we were interested in the role of ADAMTS-13 as the essential regulator of VWF on PMN functions in a mouse model of IPA. In our present work, we demonstrate that *Adamts13* gene deficient mice (*Adamts13* ^−/−^) show an increased mortality compared to wildtype mice when challenged with *A*. *fumigatus* conidia intratracheally (*i*.*t*.). This was associated with a higher fungal load in the lungs and albumin in the bronchoalveolar lavage fluid (BALF) and complement deposition as indicators of more severe tissue damage. *Adamts13* ^−/−^ mice had increased levels of inflammatory cytokines along with a decreased number of PMN in the BALF. Importantly, PMN from *Adamts13* ^−/−^ mice were fully functional in terms of the activation of effector mechanisms in response to microbial or fungal stimuli as well as killing of *A*. *fumigatus* conidia or hyphae. Interestingly, VWF deficient mice revealed no pronounced signs of inflammation and cleared IPA as effective as the wildtype controls. Thus, ADAMTS-13 deficiency causes an impaired innate immune response against *A*. *fumigatus* infections mediated by a dysregulated inflammatory response and impaired PMN recruitment to the lungs.

## Results

### Increased mortality after *A. fumigatus* infection in ADAMTS-13 deficient mice

**Figure 1:**
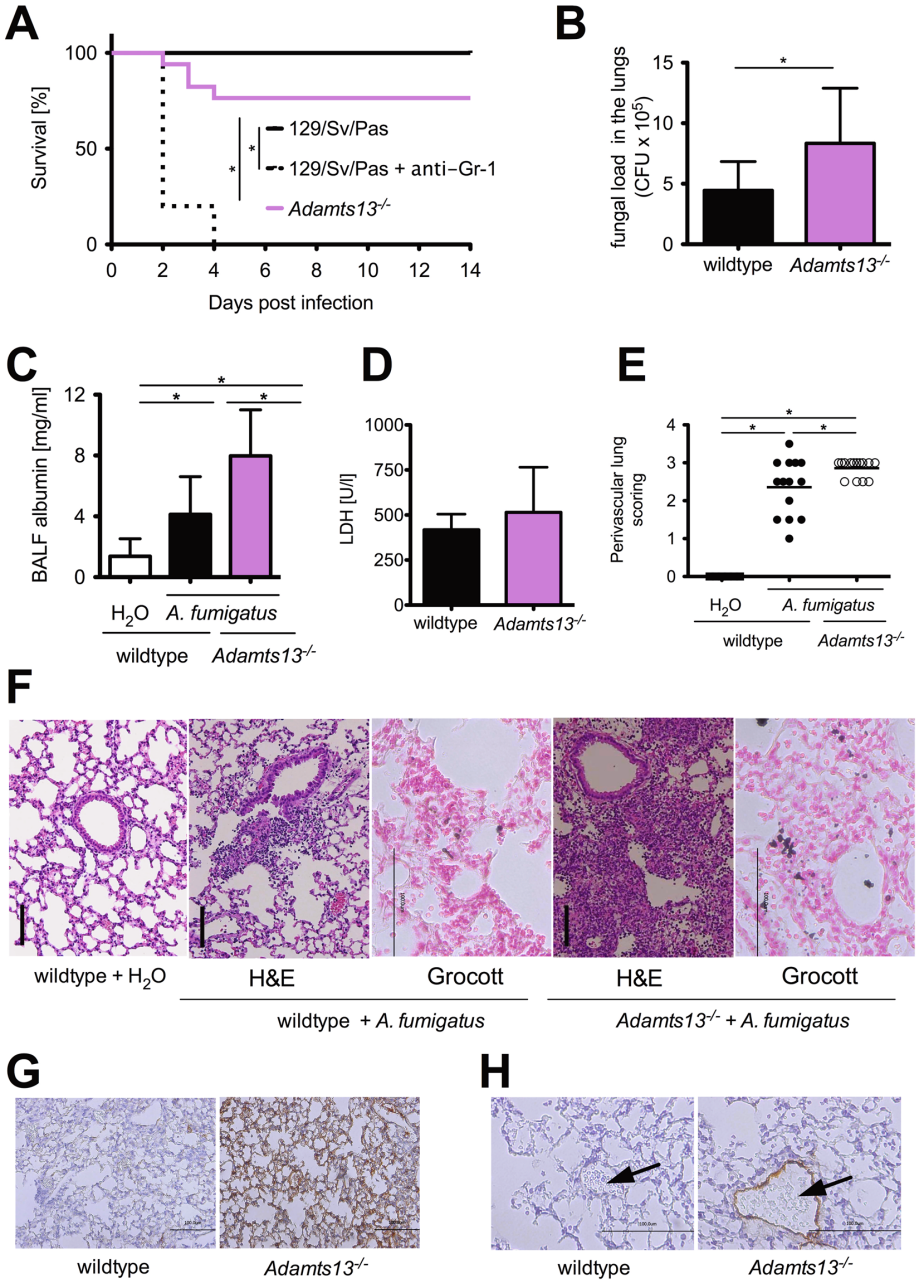
*A*. *fumigatus* infection of *Adamts13* ^−/−^ mice results in increased mortality and fungal burden of the lungs. Wildtype (129\Sv\Pas) and *Adamts13* ^−/−^ mice were infected *i*.*t*. with *A*. *fumigatus* (10^7^ conidia per animal). (**A**) Overall survival were monitored for 14 days. The cumulative results of three independent experiments (129/Sv/Pas, n = 15; 129/Sv/Pas +  Gr-1, n = 10; *Adamts13* ^−/−^, n = 17) are depicted. (*) indicates a significant difference (p < 0.05) by Mantel-Cox-Test. (**B**) 24 h after infection, some mice were sacrificed and their lungs were prepared and homogenized. The fungal burden was determined by plating serial dilutions of lung homogenates on Sabouraud 4% glucose agar plates. After 48 h the resulting colony-forming units (CFU) were enumerated. Shown are the cumulative results of three independent experiments (129/Sv/Pas, n = 10; *Adamts13* ^−/−^, n = 11) plus SD. (*) indicates a significant difference (p < 0.05) by Mann-Whitney U-test. (**C**) The albumin concentration of the BAL fluid was quantified by ELISA 24 h after infection. The cumulative results of two (H_2_O-treated 129/Sv/Pas, n = 6), three (*A*. *fumigatus*-treated Adamts13^−/−^, n = 11) or four independent experiments (*A*. *fumigatus*-treated 129/Sv/Pas, n = 13) plus SD are depicted. (**D**) Plasma LDH activity was measured 24 h after infection of wildtype (n = 4) and *Adamts13* ^−/−^ (n = 5) mice. (**E**–**H**) 24 h after infection, three mice of the indicated groups were sacrificed, paraffin sections of the lungs were prepared and stained with H&E, Grocott (**F**), C3d antibody (**G**) and VWF antibody (**H**). (**E**) Perivascular inflammation were scored by S. R. who was blinded to the experimental treatment groups. (*) indicates a significant difference (p < 0.05) by one-way ANOVA with Bonferroni’s posttest. (**F**–**H**) Representative images of lung sections are depicted (H&E, Grocott and C3d 400x magnification, VWF 800x magnification), vessels are marked by an arrow.

To assess the relevance of ADAMTS-13 and VWF in immunity against *A*. *fumigatus*, we used a mouse model of IPA. As immune competent mice are able to clear with *A*. *fumigatus* infections^5^, we infected neutropenic wildtype mice with *A*. *fumigatus* resulting in a lethal course of pneumonia in all animals within 4 days (Fig. 1A). In contrast, undepleted wildtype mice presented clinical signs of infection, but fully recovered from infection (survival rate of 100%). Interestingly, *Adamts13* ^−/−^ mice developed aggravated clinical signs of systemic infection during the first four days after inoculation indicated by weight loss, immobility and fur changes (average clinical score 4.6 in *Adamts13* ^−/−^ versus 2 in wildtype mice). This finally resulted in a mortality rate of 24% in *Adamts13* ^−/−^ mice (Fig. 1A). Analyzing the fungal burden in the lungs 24 h post infection, we detected a two fold increased fungal load in *Adamts13* ^−/−^ mice compared to the wildtype controls (Fig. 1B). In addition, we quantified alveolar albumin in the BALF 24 h after *A*. *fumigatus* exposure as a surrogate endpoint for the severity of acute lung injury and vascular leakage. As shown in Fig. 1C, infected wildtype mice had an increased albumin concentration in the BALF compared to sham treated mice, indicative of acute pulmonary injury. However in *Adamts13* ^−/−^ mice, the albumin levels in the BALF were twice as high as in the wildtype controls. To assess lung damage and cell death in this infectious condition plasma samples were analyzed for lactate dehydrogenase (LDH) activity, showing slightly elevated LDH levels in *Adamts13* ^−/−^ animals compared to wildtype mice (Fig. 1D). Together, this suggested an increased inflammatory response and lung injury along with an impaired ability to clear *A*. *fumigatus* infection in the absence of ADAMTS-13.

To verify these findings, we performed histological analyses of lung sections (Fig. 1E,F). H&E staining showed hyperemia and cellular, mainly neutrophilic infiltrates that were denser and displayed a perivascular pattern in the lungs from *Adamts13* ^−/−^ mice compared to the wildtype controls. Visualization of fungal cell walls by Grocott’s staining furthermore demonstrated invasively growing *A*. *fumigatus* hyphae in *Adamts13* ^−/−^ as well as wildtype mice (Fig. 1F). In addition, we performed immunohistochemical staining for C3d detecting more pronounced complement deposition on the whole lung tissue (Fig. 1G), as well as pronounced vascular VWF deposition (Fig. 1H) in the lungs of infected *Adamts13* ^−/−^ mice compared to the respective wildtype controls. Importantly, we were unable to detect microthrombi in the lung sections of *Adamts13* ^−/−^, nor did we observe thrombocytopenia or hemolysis indicative of TTP, which is in line with previous observations that ADAMTS-13 deficiency is not sufficient to induce TTP in mice^20^.

Taken together, this set of data clearly shows that ADAMTS-13 deficiency is associated with an increased susceptibility to fungal infections resulting in a higher mortality rate, apparently leading to an uncoordinated inflammatory response along with a decreased ability to clear the fungal burden.

### ADAMTS-13 deficiency results in impaired recruitment of PMN after infection with *A. fumigatus*

For fungal clearance, an adequate recruitment and activation of PMN is essential^5^. To elucidate whether the diminished fungal clearance is a result of an impaired inflammatory response, we analyzed the peripheral blood and BALF of wildtype and *Adamts13* ^−/−^ mice 24 h after *in vivo* exposure to *A*. *fumigatus*.

**Figure 2:**
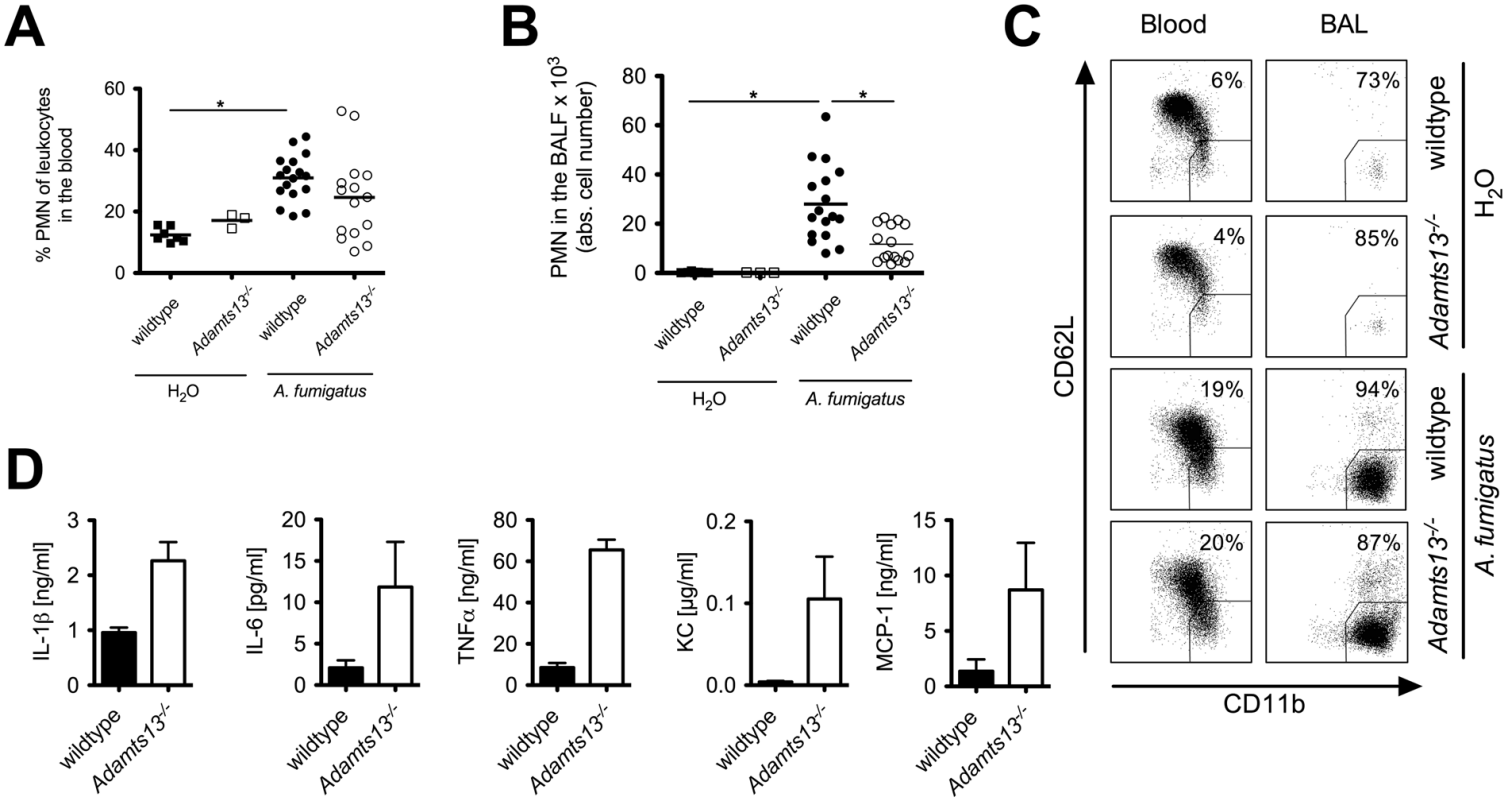
PMN-recruitment in *Adamt13* ^−/−^ mice is impaired after infection with *A*. *fumigatus*. Wildtype (129/Sv/Pas) or *Adamts13* ^−/−^ mice were infected *i*.*t*. with *A*. *fumigatus* (10^7^ conidia per animal) or H_2_O (sham control). Peripheral blood and BAL fluid were collected 24 h after infection and analyzed by flow cytometry. (**A**,**B**) The frequency of PMN (CD11b^high^ Gr-1^+^) in the peripheral blood (**A**) and total PMN counts in the BAL fluid (**B**) are shown. Depicted are the cumulative results of three (H_2_O-treated 129/Sv/Pas, n = 7), one (H_2_O-treated *Adamts13* ^−/−^, n = 3) or four independent experiments (*A*. *fumigatus*-treated 129/Sv/Pas, n = 17; *Adamts13* ^−/−^, n = 16) plus SD. (**C**) For analysis of PMN phenotype in blood or BAL fluid, CD62L shedding and CD11b regulation was quantified gating on Gr-1^+^ cells. The percentage of activated PMN (Gr-1^+^ CD11b^high^ CD62L^low^ cells) of a representative sample is shown. (*) indicates a significant difference (p < 0.05) by one-way ANOVA with Bonferroni’s posttest. (**D**) Different cytokines and chemokines in the BAL fluid of infected mice were quantified by Bioplex-Assay. The cumulative results of one experiment (n = 3) out of two are shown. Mann-Whitney U-test calculated no significant differences between knockout and wildtype animals.

As shown in Fig. 2A in peripheral blood of infected wildtype and *Adamts13* ^−/−^ mice, we observed an increased percentage of PMN compared to sham treated mice. This increased absolute neutrophil count (ANC) was not significantly different in wildtype and *Adamts13* ^−/−^ mice. In addition, we found a significantly increased number of PMN in the BALF of infected wildtype mice compared to sham control (Fig. 2B), indicative of the local recruitment of PMN to the site of inflammation. In contrast, we found a significantly reduced number of PMN in the BALF of infected *Adamts13* ^−/−^ mice. Together with the increased density of PMN tissue infiltrates (Fig. 1F), this suggests an impaired PMN recruitment compared to wildtype animals and a possible explanation for the previously observed increased fungal load in *Adamts13* ^−/−^ mice.

To address whether the impaired ability to clear the *A*. *fumigatus* infection in the absence of ADAMTS-13 was due to a defective PMN activation, we assessed the PMN activation status *in vivo* during infection, using the upregulation of CD11b for degranulation and shedding of CD62L as markers. Both markers are constitutively expressed on resting PMN. CD11b is mobilized from specific granules to the cell surface, while CD62L is rapidly down regulated by enzymatic shedding in activated PMN. In the peripheral blood of infected wildtype or *Adamts13* ^−/−^ mice (Fig. 2C), we were unable to detect significant differences. Comparing the local PMN activation status in the BALF with the peripheral blood, we detected a strong upregulation of CD11b and a concurrent loss of CD62L expression in the lungs of infected wildtype and *Adamts13* ^−/−^ mice, respectively. Once again, there were no differences between infected wildtype and *Adamts13* ^−/−^ mice, suggesting that there are no functional PMN defects impeding the antifungal immune response, at least analyzing CD11b and CD62L shedding.

To characterize the inflammatory response upon *A*. *fumigatus* infection more closely, we quantified inflammatory mediators in infected wildtype and *Adamts13* ^−/−^ mice. As depicted in Fig. 2D, increased levels of the cytokines IL-1β, IL-6, IL-10 and TNFα as well as IL-1α, IL-2, IL-4, IFN-γ and G-CSF (data not shown) were detected in *A*. *fumigatus* infected *Adamts13* ^−/−^ mice compared to the wildtype controls. In addition, the chemokines KC and MCP-1 as well as MIP-1α and MIP-1β (data not shown) were strongly elevated in infected *Adamts13* ^−/−^ mice indicating an increased inflammatory response and excluding that a defective chemokine production is responsible for the diminished PMN recruitment observed in *Adamts13* ^−/−^ mice.

Taken together, our results show a dense PMN infiltration in the lung tissue, but a delay in PMN recruitment to the BALF of *Adamts13* ^−/−^ mice. Notably, there is no apparent defect in PMN activation along with an increased inflammatory cytokine response after infection with *A*. *fumigatus* in *Adamts13* ^−/−^ mice.

### PMN effector functions are not affected in response to *A. fumigatus* in ADAMTS-13 deficient mice

To clarify whether PMN from *Adamts13* ^−/−^ mice have a general impairment in their effector functions, we incubated PMN from *Adamts13* ^−/−^ mice or the respective wildtype controls *in vitro* with phorbol ester (PMA) or the formyl peptide agonist WKYMVm and analyzed phagocytosis, degranulation via CD11b expression, CD62L shedding, the oxidative burst and apoptosis.

**Figure 3:**
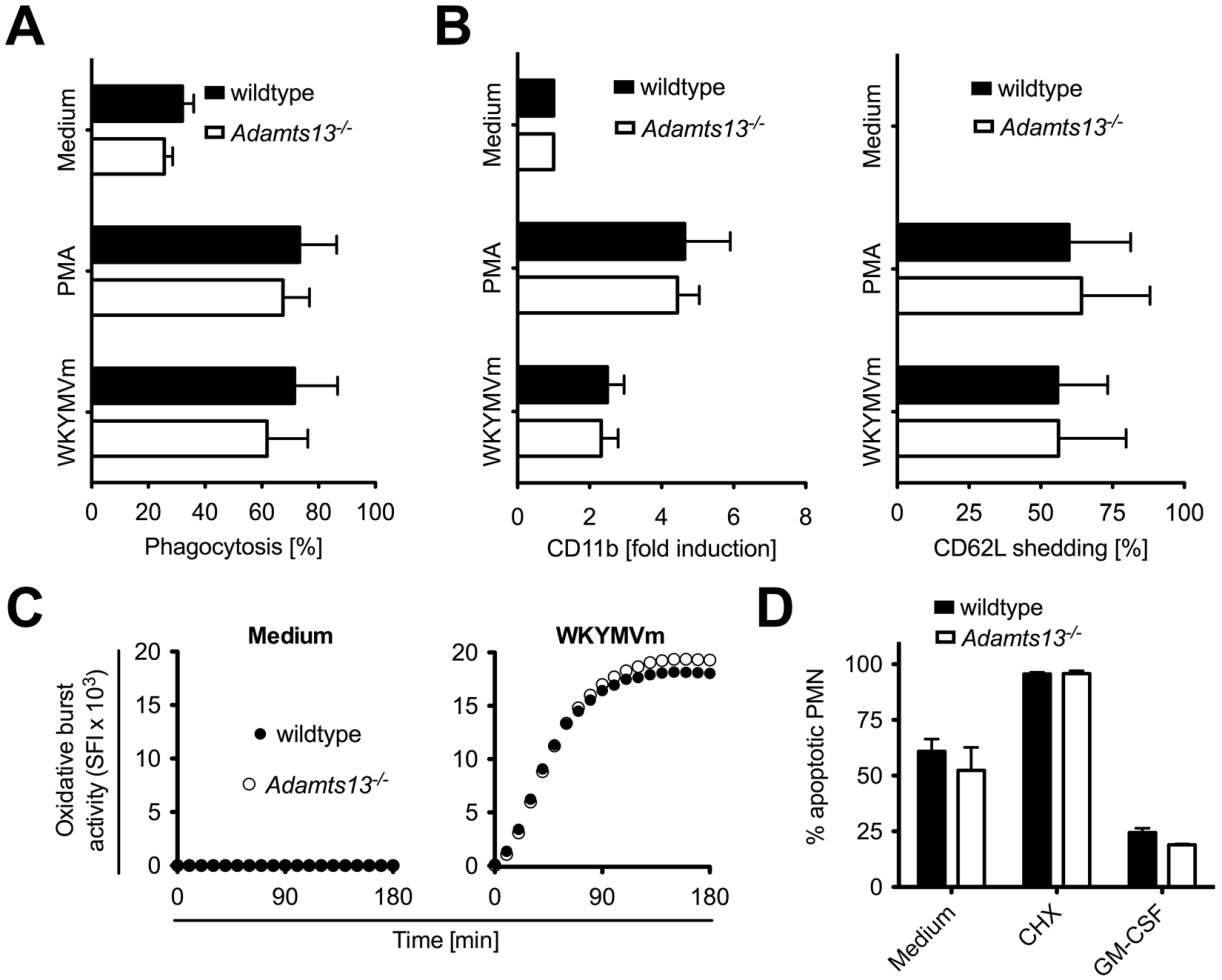
PMN from *Adamts13* ^−/−^ mice display normal activation patterns. PMN (10^6^/ml) derived from wildtype (129/Sv/Pas) or *Adamts13* ^−/−^ mice were stimulated as indicated for 4 h. (**A**) Phagocytosis was determined in absence or presence of PMA (50 ng/ml) or formyl-peptide agonist WKYMVm (8 µg/ml) using PE-labeled polystyrene beads (5.7 × 10^7^ particles/ml). After 4 h PMN were analyzed by flow cytometry. (**B**) PMN were stimulated as in (**A**) and after 4 h expression of CD11b and CD62L was measured by flow cytometry gating on Ly-6G^+^ cells. (**C**) The oxidative burst activity was quantified with the fluorogenic dye DCFH-DA that converts into green fluorescent DCF in the presence of reactive oxygen species. Untreated (medium) or with formyl-peptide agonist WKYMVm (8 µg/ml) stimulated PMN were analyzed in a fluorescence reader over time. (**D**) Apoptosis of PMN was determined with Nicoletti assay after 24 h in absence or presence of cycloheximide (CHX, 10 µg/ml) and GM-CSF (50 ng/ml). The summarized results of tow independent experiments (129/Sv/Pas and *Adamts13* ^−/−^, n = 5) plus SD are shown. A one-way ANOVA with Bonferroni’s posttest detects no significant difference (p < 0.05) between PMN from wildtype and *Adamts13* ^−/−^ mice.

As shown in Fig. 3A, the phagocytosis of polystyrene beads was strongly increased by PMA or WKYMVm. Moreover, stimulation with PMA or WKYMVm resulted in strong upregulation of CD11b and CD62L shedding (Fig. 3B) whereas WKYMVm induced a strong oxidative burst (Fig. 3C). However, we were unable to detect any significant differences between PMN from wildtype or *Adamts13* ^−/−^ mice.

Next, we examined the induction of apoptosis, as PMN are short-lived cells known to be highly sensitive to external stimuli and undergo apoptosis rapidly unless adequately activated. As depicted in Fig. 3D, about 50% of untreated PMN were apoptotic after 24 h. Incubation with cycloheximide as strong inducer of apoptosis^21^ resulted in nearly 100% apoptosis, while stimulation with GM-CSF prolonged the survival of PMN^22^. Again, no significant differences between both mouse strains were detected indicating that ADAMTS-13 deficiency does not impair PMN functionality in general.

**Figure 4:**
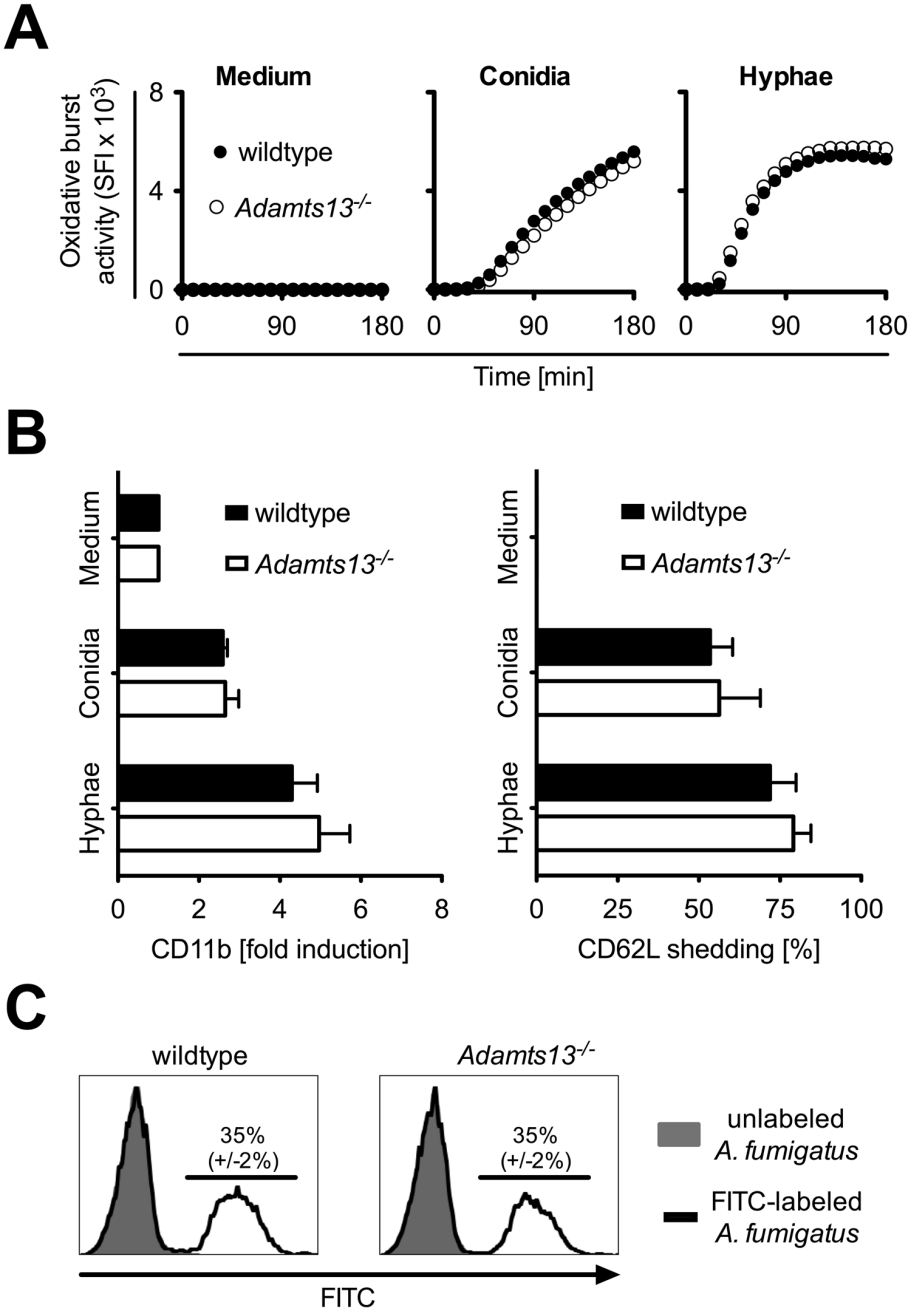
PMN from *Adamts13* ^−/−^ mice show no defect in activation or phagocytosis after contact with *A*. *fumigatus*. PMN (10^6^/ml) derived from wildtype (129/Sv/Pas) or *Adamts13* ^−/−^ mice were stimulated with *A*. *fumigatus* conidia (10^6^ conidia/ml) or conidia grown for 16 h to build hyphae (10^6^ conidia/ml). (**A**) The oxidative burst activity was assessed as described above (in Fig. 3C). (**B**) The expression of CD11b and CD62L was determined by flow cytometry gating on Ly6G^+^ cells after 4 h and normalized to untreated cells (medium). Depicted are the cumulative results of two independent experiments plus SD (129/Sv/Pas and *Adamts13* ^−/−^, n = 5). (**C**) After 1 h incubation with FITC-labeled *A*. *fumigatus* conidia (10^6^ conidia/ml) phagocytosis was determined by flow cytometry. The uptake of unlabeled conidia was used as negative control. One representative experiment performed in triplicates of two independent experiments is shown. A one-way ANOVA with Bonferroni’s posttest detects no significant difference (p < 0.05) between PMN from wildtype and *Adamts13* ^−/−^ mice.

To further clarify whether other activation pathways important for fungal recognition and clearance were fully functional in PMN derived from *Adamts13* ^−/−^ mice, we used *A*. *fumigatus* conidia and hyphae for stimulation and analyzed PMN effector mechanisms as before. Once again, PMN from wildtype or *Adamts13* ^−/−^ mice showed a comparable induction of the oxidative burst activity after stimulation with *A*. *fumigatus* conidia or hyphae (Fig. 4A). In addition, both conidia as well as hyphae caused a strong upregulation of CD11b and shedding of CD62L in PMN (Fig. 4B) while no differences between PMN from wildtype or *Adamts13* ^−/−^ mice were detectable. To analyze the phagocytosis of *A*. *fumigatus* conidia, conidia were labeled with FITC, and the uptake was quantified by flow cytometry (Fig. 4C) detecting a comparable phagocytosis of PMN from wildtype or *Adamts13* ^−/−^ mice.

Overall, these data clearly demonstrate that effector functions of PMN are not affected in ADAMTS-13 deficient mice neither in general nor in response to *A*. *fumigatus* conidia or hyphae.

### PMN mediated killing of *A. fumigatus in vitro* is not impaired in ADAMTS-13 deficient mice

**Figure 5:**
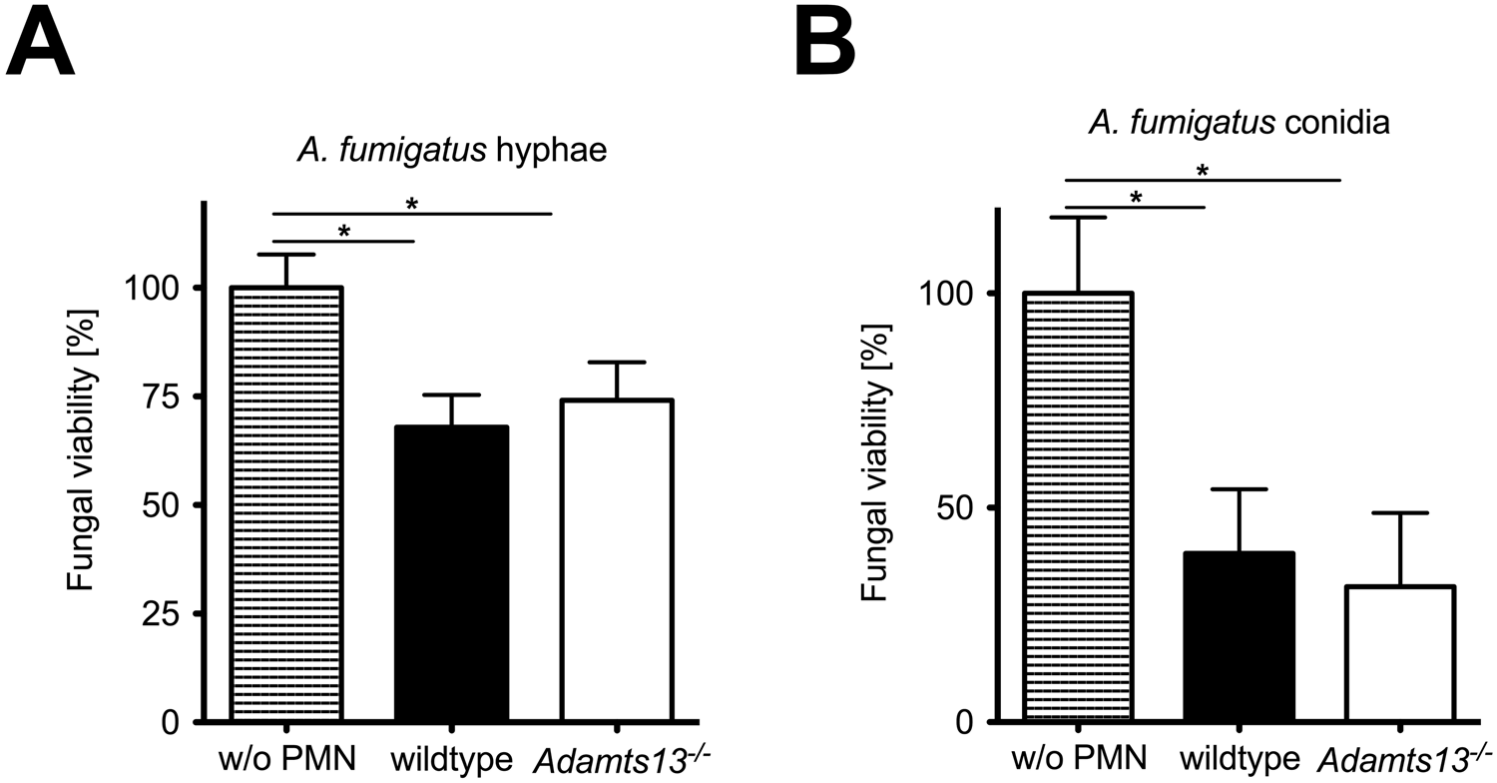
Killing of *A*. *fumigatus* conidia or hyphae is not diminished in PMN from *Adamts13* ^−/−^ mice. Killing of A. *fumigatus in vitro* by PMN (10^6^ cells/ml) was assessed by modified XTT assay. After 4 h co-culture with conidia (3 × 10^3^/ml) (**A**) or conidia (3 × 10^3^/ml) germinated for 16 h (**B**), PMN were lysed and conidia were grown for further 16 h. The viability of hyphae was determined using a colorimetric XTT assay. Depicted is the mean plus SD of one representative experiment performed in triplicates out of two. (*) indicates a significant difference (p < 0.05) by one-way ANOVA with Bonferroni’s posttest.

While the effector functions of PMN from *Adamts13* ^*−/−*^ mice were not impaired, we analyzed whether there might be a direct impact of ADAMTS-13 on fungal killing by PMN. PMN were co-cultured with *A*. *fumigatus* conidia or hyphae and the viability of germinated fungus were determined using a colorimetric assay, subsequently. As shown in Fig. 5, we observed a comparable killing of conidia (Fig. 5A) and hyphae (Fig. 5B) by PMN from wildtype and *Adamts13* ^−/−^ mice indicating that also this effector function is not impaired through ADAMTS-13 deficiency.

### *A. fumigatus*-activated serum from *Adamts13*^−/−^ mice has unaltered chemotactic ability on human PMN

**Figure 6:**
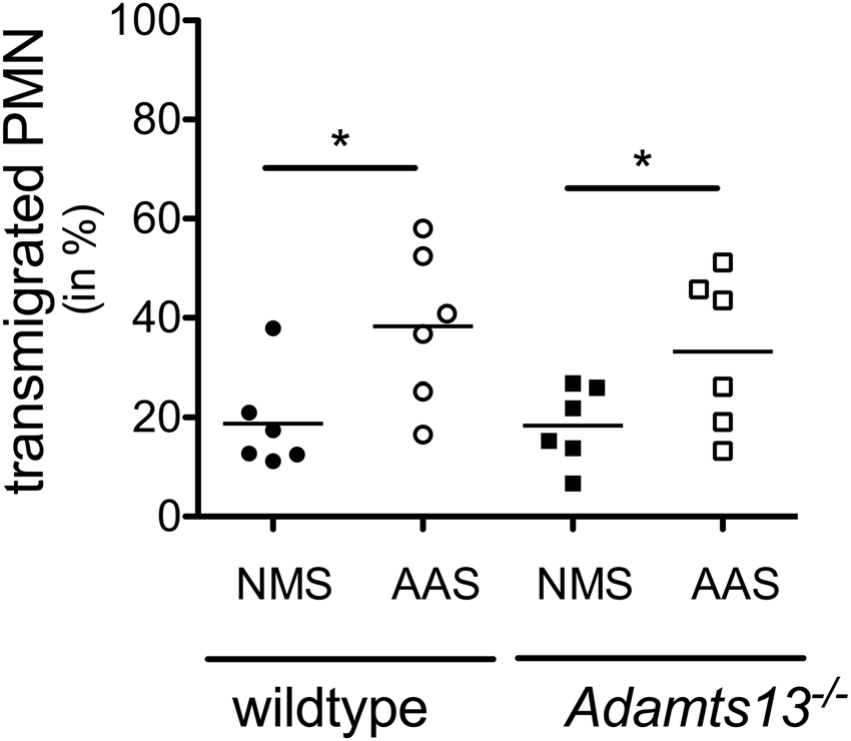
*A*. *fumigatus* activated serum from *Adamts13* ^−/−^ mice does not alter PMN migration. Serum from wildtype (129/Sv/Pas) or *Adamts13* ^−/−^ mice was activated with 10^8^/ml *A*. *fumigatus* conidia (**A**). fumigatus activated serum; AAS) and analyzed for chemotactic ability. For transwell migration of human PMN from healthy donors towards AAS or non-activated mouse serum (NMS), serum was added to the lower chambers and calcein labeled PMN (2 × 10^5^ cells) in upper wells and incubated for 1 h at 37 °C. The cumulative results of six independent experiments are depicted. By two-tailed Wilcoxon signed rank test significant increase of migration is shown for *A*. *fumigatus*-activated serum compared to native serum in both mice types. Statistical significant differences of PMN migration towards activated serum between *Adamts13* ^−/−^ mice compared to wildtype sera were not observed.

Since UL-VWF multimers have no effect on C3b cleavage and permit default complement activation compared to normal plasma VWF multimers^23^, we investigated whether altered serum complement content and therefore the chemotactic ability of serum components is involved in modified PMN migration. While PMN from *Adamts13* ^−/−^ mice showed normal effector functions, we exclusively analyzed *Adamts13* ^−/−^ mouse serum and migration towards activated complement by employing PMN from healthy human donors in this assay. Serum of *Adamts13* ^−/−^ and wildtype mice was incubated with *A*. *fumigatus* to provide complement activation, and PMN migration towards activated and non-activated serum was assessed by transwell experiment. Using sera of both mouse strains a significant increase of migration was observed towards *A*. *fumigatus*-activated serum as compared to native serum (Fig. 6). No significant impairment of PMN migration towards activated serum of *Adamts13* ^−/−^ mice compared to wildtype serum was observed. However, this experimental setup rather addresses the role of UL-VWF on complement activation and not on PMN migration. Therefore, we conducted a set of experiments where *Adamts13* ^−/−^ mouse plasma (containing UL-VWF) or wildtype plasma was coated on the transwell membrane and PMN were added afterwards for the transwell migration assay. Also in this experimental setup, we were unable to detect any differences (data not shown). These results indicate that not only PMN and UL-VWF control cell influx in IPA, but suggest a more complex cellular interaction, most likely also involving endothelial cells and platelets.

### VWF deficiency does not impair inflammatory response in IPA

**Figure 7:**
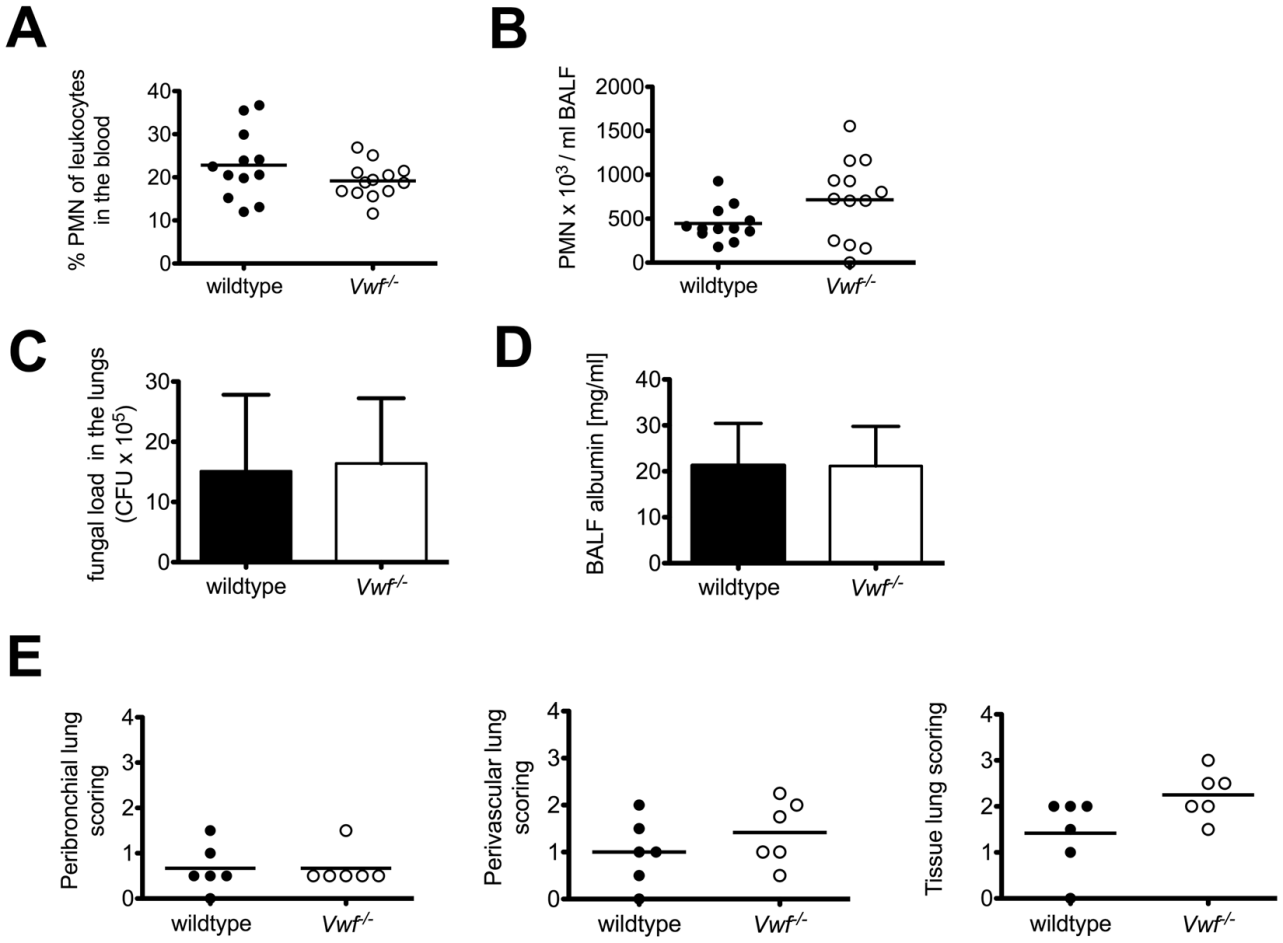
VWF deficiency does not impair inflammatory response in IPA. Inflammatory response to IPA was investigated in wildtype (B6) and *Vwf* ^−/−^ mice 24 h after infection. ANC in the blood (**A**) and in the BALF (**B**) was measured by an animal blood counter. Shown are the cumulative results of two independent experiments (B6, n = 12; *Vwf* ^−/−^, n = 13). (**C**) The fungal burden was determined by plating serial dilutions of lung homogenates on Sabouraud 4% glucose agar plates. After 48 h CFU were enumerated. Shown are the cumulative results of two independent experiments (B6, n = 12; *Vwf* ^−/−^, n = 13) plus SD. (**D**) The albumin concentration of the BAL fluid was quantified by ELISA. The cumulative results of two independent experiments (B6, n = 9; *Vwf* ^−/−^, n = 10) plus SD are depicted. (**E**) 6 mice of the indicated groups were sacrificed and paraffin sections of the lungs were prepared and stained with H&E. Peribronchial, perivascular and tissue lung scores were determined for the indicated groups by H. B. who was blinded to the experimental treatment groups.

So far VWF is known to be the exclusive target of the metalloprotease ADAMTS-13. Therefore, we were interested if the observed effects in impaired inflammatory response to IPA can also be detected in the context of VWF deficiency. IPA was induced in *Vwf* ^−/−^ and wildtype (B6) mice as described above. 24 h after infection with *A*. *fumigatus* VWF deficient and wildtype mice showed comparable ANCs in the blood (Fig. 7A). In contrast to our findings in *Adamts13* ^*−*/*−*^ mice, VWF deficiency was not associated with a dysregulated recruitment of neutrophils to the lungs, as analyzed by neutrophil counts in the BALF (Fig. 7B).

To assess the severity of the pulmonary *A*. *fumigatus* infection, lung homogenates were analyzed for fungal load. After cultivation for 48 h on Sabouraud 4% glucose agar, samples from wildtype and *Vwf* ^−/−^ mice showed comparable pulmonary fungal burdens (Fig. 7C). Along with this, there were no significant differences in lung injury, represented by the albumin amount in the BALF (Fig. 7D) or leukocyte infiltration by histology scored as perivascular, peribronchial or total tissue infiltration (Fig. 7E).

These results show that VWF is not required for the coordinated inflammatory response and in particular for PMN recruitment to the lungs in IPA. Nevertheless, together with our data from *Adamts13* ^−/−^ mice our results suggest that VWF multimers and their regulation by ADAMTS-13 are important for the regulation of PMN extravasation in IPA.

## Discussion

In this study, we demonstrate that the worse outcome of *Adamts13* ^−/−^ mice in IPA is associated with a higher fungal load in the lungs, more severe tissue damage and increased levels of inflammatory cytokines indicating a dysbalanced inflammatory response and compatible with the clinical signs of sepsis. Interestingly, in contrast to the enhanced cytokine concentration and leukocyte infiltrates in the lungs, we detected diminished PMN migration in BALF in the absence of ADAMTS-13 indicating an important function of this protease in the regulation of PMN recruitment to the infectious site. However, PMN from *Adamts13* ^−/−^ mice were fully functional in terms of the activation of effector mechanisms in response to microbial or fungal stimuli as well as killing of *A*. *fumigatus* conidia or hyphae. It needs to be mentioned that these experiments were performed with bone marrow derived PMNs as only this method allows to obtain purified PMNs in sufficient numbers and least pre-activated by the isolation procedure. However, these experiments do not address functional differences in PMN activation *in vivo*, e.g. in the peripheral blood, at the endothelial surface or within the lung tissues. Therefore, due to the technical limitations we cannot exclude that there are functional differences in PMN activation in *Adamts13* ^−/−^ mice *in vivo* that we are unable to detect *in vitro*.

In inflammatory conditions such as endotoxemia and sepsis increased levels of UL-VWF and decreased activity of ADAMTS-13 are found implicating a general role in inflammatory responses^16, 17^. Besides, immune paralysis i.e. as in sepsis increases the susceptibility for IPA^19^, but the underlying mechanisms are currently not clear. To investigate the relevance of ADAMTS-13 deficiency in this context, we employed a mouse model of IPA and demonstrate that ADAMTS-13 deficiency is associated with increased mortality and increased signs of inflammation in IPA. Remarkably, analyzing the inflammatory response to IPA in the absence of VWF, VWF deficiency had no influence on fungal load or lung damage. Thus, VWF is not directly required for cell recruitment, but since VWF is the only known substrate for ADAMTS-13, our data allow the conclusion that the regulation in size of UL-VWF multimers by ADAMTS-13 is an important mechanism to control invasion of PMNs in inflamed lung tissue. This is in line with other findings demonstrating an improved survival in patients with severe sepsis with higher levels of ADAMTS-13 activity^24^. Furthermore, in inflammation-associated ADAMTS-13 deficiency, a marked imbalance between ADAMTS-13 activity and VWF antigen level are associated with the appearance of UL-VWF multimers in plasma, correlating with organ dysfunction and lethality^25^. In contrast, others have demonstrated that VWF parameters are reciprocally correlated with ADAMTS-13 activity in severe sepsis and septic shock without prognostic value regarding the outcome^26^. Moreover, in a mouse model of sepsis, VWF secretion was a major determinant of ADAMTS-13 decrease and played an important role in sepsis-induced mortality. However, the complete absence of ADAMTS-13 had no detectable impact in this sepsis model and increased ADAMTS-13 activity had no impact on the survival rate^27^. Later, it has been shown that association of functional deficiency of ADAMTS-13 and septic shock is independent of disseminated intravascular coagulation (DIC) and that ADAMTS-13 functional deficiency is a prognostic factor for mortality in septic shock patients^28^.

Depending on their size, VWF multimers shut down complement activation by acting as a cofactor for Factor I-mediated cleavage of complement C3b. Therefore normal plasma VWF multimers prevent complement activation and promote generation of inactivated C3b (iC3b) whereas UL-VWF multimers have no effect on C3b cleavage and fail to inactivate complement^23^. Since PMN showed to be functionally fully intact in the absence of ADAMTS-13, and also given the increased complement deposition in the lungs of *Adamts13* ^−/−^ mice, we investigated whether activated complement is involved as a chemotactic factor in PMN recruitment^29^, detecting no impaired PMN migration towards *A*. *fumigatus*-activated sera of *Adamts13* ^−/−^ mice or when *Adamts13* ^−/−^ plasma was coated to the transwell membrane. We hypothesize that the regulation in size of VWF by ADAMTS-13 controls the formation of VWF binding to platelets and endothelium, which in turn controls transendothelial PMN migration. This notion is supported by Petri *et al*. where the authors demonstrate that VWF associated platelets are important for PMN extravasation in a peritonitis model^18^. Although not directly addressed, their results showing that platelet depletion inhibits PMN recruitment to the peritoneum are in support of this notion, as UL-VWF in *Adamts13* ^−/−^ facilitates platelet-rich thrombus formation^20^. Furthermore data from our group demonstrates that PMN are activated by platelets via triggering receptor expressed on myeloid cells 1 (TREM-1)^30^ allowing the assumption that PMN may become prematurely activated during the migration process causing excessive tissue damage. Interestingly, TREM-1 has also been described to be of relevant for transendothelial PMN migration in a model of *Pseudomonas* pneumonia^31^. However, due to the complexity of the proposed interactions and limitation of experimental methods to replicate such a complex scenario of transendothelial PMN migration in the presence of activated endothelial cells and platelets *in vitro*, we have to leave the definitive proof of the underlying mechanism open. Whether enhanced complement activation in ADAMTS-13 deficient environment^23^ or other factors such as HMGB-1^32^ are the underlying mechanisms for the poorer outcome of *Adamts13* ^−/−^ mice in our IPA model, is currently not clear and needs further investigations. Furthermore the finding that ADAMTS-13 deficiency, but not VWF deficiency impairs inflammatory response still leaves the question unanswered, whether ADAMTS-13 has another function besides cleaving VWF, or if this is caused by UL-VWF multimers.

In conclusion, we demonstrate an important role for ADAMTS-13 in the innate immune response against *A*. *fumigatus* induced pneumonia. ADAMTS-13 is required to suppress hyper-inflammatory responses in IPA. While ADAMTS-13 is not involved in the activation of PMN effector functions, it causes a delayed PMN recruitment to the lungs leading to increased tissue damage, most likely by interaction with adhesion at the endothelial interface.

## Materials and Methods

### Mice and fungal strain

*Adamts13* ^−/−^ mice were on 129/Sv/Pas background^20^ and *Vwf* ^−/−^ mice were on B6 background^33^. All animal procedures were performed in accordance with the institutional guidelines and approved by the responsible national authority (National Investigation Office Rheinland-Pfalz, Approval ID: AZ 23 177-07/G11-1-034).

The *A*. *fumigatus* strain ATCC 46645 was kindly provided by M. Gunzer (Molecular Immunology, University of Duisburg-Essen, Germany) and cultured in *Aspergillus* minimal medium^34^.

### Mouse model of IPA

Mice were anesthetized and received 10^7^ *A*. *fumigatus* conidia *i*.*t*. as described previously^5^.

#### Survival

Severity of systemic infection was daily examined by the evaluation of weight, activity, posture, skin and fur appearance as previously described, and overall survival was monitored for 14 days^35^. Where indicated, PMN depletion was induced by *i*.*p*. injection of anti-Gr-1 antibody (150 µg, clone RB6-8C5) one day before infection.

#### Severity of infection

Mice were sacrificed 24 h after infection. Paraffin embedded lung sections were stained with hematoxylin and eosin (H&E) to assess inflammatory responses, Grocott-Gomori silver stain to visualize fungi, mouse complement component C3d antibody (R&D systems) and VWF antibody (Dako). For analysis of inflammation H&E-stained tissue sections were examined in blinded fashion for peribronchial, perivascular and tissue inflammation, scored on a scale from 0 to 4^36^. For analysis of blood and BALF, blood samples were collected and lungs were flushed with 1 ml PBS. Cells in the blood and BALF were analyzed by flow cytometry or an animal blood counter (scil animal care company) respectively. Albumin concentration in the BALF was quantified by standard enzyme-linked immunosorbent assay (ELISA) (Bethyl Laboratories). Plasma LDH measurement was kindly provided by a standard assay of the central laboratory facility of the University Medical Center Mainz. For the detection of inflammatory mediators, ƒbead-based immunoassays were used (Bio-Plex Pro mouse cytokine bead-based immunoassay; Bio-Rad). The results were quantified by the Luminex xMAP/Bio-Plex 200 System with Bio-Plex Manager Software 5.0^21^. To characterize *in vivo* fungal burden^5^, lungs were homogenized mechanically and serial dilutions were plated on Sabouraud 4% glucose agar (Carl Roth). After 48 h colony-forming units (CFU) were enumerated.

### Analysis of PMN functions in vitro

PMN were enriched from the bone marrow of mice by magnetic cell sorting using biotin labeled Ly-6G/C specific antibodies (clone RB6-8C5) and SA-beads (Miltenyi)^5^. PMN were treated as indicated and analyzed by flow cytometry. The mean fluorescence intensity (MFI) of the indicated markers were standardized to untreated PMN. Phagocytosis was evaluated using PE-labeled polystyrene microspheres (diameter 1 µm, Fluoresbrite Plain Microspheres PCRed, Polysciences)^5^. Phagocytosis of *A*. *fumigatus* conidia was evaluated using FITC-labeled spores^35^. Oxidative burst activity was detected by reactive oxygen intermediates that oxidized non-fluorescent dichloro-fluorescein diacetate (DCFH-DA, Sigma-Aldrich) into green fluorescent DCF^30^. Kinetics were measured with a fluorescence reader (SpectraFluor 4)^5^. To obtain the specific fluorescence index (SFI), the background fluorescence of untreated, labeled cells was subtracted. Apoptosis was evaluated by detection of DNA fragmentation, quantifying hypodiploid nuclei according to a modified protocol described previously^22^. For fungal killing *in vitro*, killing of conidia and hyphae was determined using a modified XTT assay^5^.

### Serum activation and PMN migration

All human studies were performed after obtaining written consent from healthy volunteer donors in accordance with the Declaration of Helsinki and were approved by the Landesaerztekammer Rhineland-Palatine Ethics Committee according to the institutional guidelines. Chemotactic serum contents were analyzed by migration of healthy human PMN towards *A*. *fumigatus*-activated *Adamts13* ^−/−^ and corresponding 129/Sv/Pas mice sera. PMN were isolated from heparinized whole blood samples, processed by dextran sedimentation and Histopaque® centrifugation (Histopaque®-1077, Sigma-Aldrich) and cells were calcein-AM labeled (Life Technologies GmbH). Mice sera were activated by incubation with *A*. *fumigatus* conidia. Migration was provided using a Corning® HTS Transwell®-96 well permeable support (3 µm) (Sigma-Aldrich). For plasma preparation mice were deeply anesthetized and samples of citrate plasma were gained by cardiac puncture. Citrate plasma was coated on upper wells (1 h, 37 °C) for respective experiments.

### Statistical analysis

Statistical analyses were performed using GraphPad Prism (version 5.0a for Mac OS X, GraphPad Software). For comparison between two groups a Mann-Whitney U-test was used. Comparisons of multiple groups were performed by one-way ANOVA with Bonferroni’s posttest. For all analyses, p < 0.05 was considered as statistically significant.
