# Author Correction: ADAMTS-13 regulates neutrophil recruitment in a mouse model of invasive pulmonary aspergillosis

**DOI:** 10.1038/s41598-018-32187-7

**Published:** 2018-09-26

**Authors:** Astrid Alflen, Steve Prüfer, Katharina Ebner, Sebastian Reuter, Pamela Aranda Lopez, Inge Scharrer, Fumiaki Banno, Michael Stassen, Hansjörg Schild, Kerstin Jurk, Markus Bosmann, Hendrik Beckert, Markus P. Radsak

**Affiliations:** 1 IIIrd Dept. of Medicine, Johannes Gutenberg-University Medical Center, Johannes Gutenberg-University, Mainz, Germany; 2 Institute for Immunology, Johannes Gutenberg-University Medical Center, Johannes Gutenberg-University, Mainz, Germany; 3 Center for Thrombosis and Hemostasis, Johannes Gutenberg-University Medical Center, Johannes Gutenberg-University, Mainz, Germany; 4 Department of Molecular Pathogenesis, National Cerebral and Cardiovascular Center, Suita, Japan

Correction to: *Scientific Reports* 10.1038/s41598-017-07340-3, published online 03 August 2017

This Article contains an error in the Results section under subheading ‘PMN mediated killing of *A*. *fumigatus in vitro* is not impaired in ADAMTS-13 deficient mice’.

“As shown in Fig. 5, we observed a comparable killing of conidia (Fig. 5A) and hyphae (Fig. 5B) by PMN from wildtype and *Adamts13*^−/−^ mice indicating that also this effector function is not impaired through ADAMTS-13 deficiency.”

should read:

“As shown in Fig. 5, we observed a comparable killing of conidia (Fig. 5B) and hyphae (Fig. 5A) by PMN from wildtype and *Adamts13*^−/−^ mice indicating that also this effector function is not impaired through ADAMTS-13 deficiency.”

